# MMP16 is a marker of poor prognosis in gastric cancer promoting proliferation and invasion

**DOI:** 10.18632/oncotarget.10177

**Published:** 2016-06-20

**Authors:** Li Cao, Chaowu Chen, Haihang Zhu, Xuewen Gu, Denghao Deng, Xiuchun Tian, Jun Liu, Qin Xiao

**Affiliations:** ^1^ Department of Pathology, Clinical Medical College of Yangzhou University, Subei People's Hospital of Jiangsu Province, Yangzhou, Jiangsu 225001, P.R. China; ^2^ Department of Gastroenterology, Clinical Medical College of Yangzhou University, Subei People's Hospital of Jiangsu Province, Yangzhou, Jiangsu 225001, P.R. China

**Keywords:** gastric cancer, MMP16, survival analysis, proliferation, invasion

## Abstract

Matrix metalloproteinases (MMPs) are closely associated with tumor proliferation, invasion and metastasis. In this study, we determined the MMPs expression and their clinical significances in gastric cancer (GC). We first extensive studied MMPs expression in GC in The Cancer Genome Atlas (TCGA) RNA sequence database and found MMP16 was candidate biomarker in GC. Then we validated clinical significance of MMP16 mRNA expression in 167 GC by RT-PCR. Survival analysis showed that high expression of MMP16 indicated poor overall and disease free survival (P<0.001). The proliferation and invasion potential of GC cells were determined by CCK8, colony formation and Transwell assays. Silencing of MMP16 expression significantly decreased the invasion and proliferation capacity of GC cells (P<0.05). In conclusion, MMP16 was highly expressed and correlated with poor prognosis in GC patients by promoting proliferation and invasion of GC cells. MMP16 could be a novel molecular target and prognostic marker for GC.

## INTRODUCTION

Gastric cancer (GC) remains one of the most common of cancer related mortality in East Asia, and about percentage of the cases is in China [1, 2]. The only curative treatment option for GC patients is surgical resection [2]. Although there have been advances in diagnosis and management, most GC patients present with locally advanced or metastatic disease with a 5-year survival rate of <10%. [3, 4]. The tumor-node-metastasis (TNM) staging mainly focuses on the tumor itself, that is, its biological behavior, and is the most important prognostic factor for GC. However, the current staging system is not precise for predicting patient outcomes because the prognosis varies in patients with the same disease stage. Thus, the search of biomarkers that can be used to predict of survival would be very urgent in GC [2].

Matrix metalloproteinases (MMPs), a family of zinc-dependent endopeptidases [5], are closely associated with the abilities of proliferation, invasion and metastasis in tumors [6, 7]. MMPs can promote the formation of tumor blood vessels and can degrade the extracellular matrix (ECM), [8]. MMPs family share a relative large percentage of common structural and functional areas, but differ in their substrate specificities [9]. MMPs play critical roles both in physiological process [10, 11] and in pathological processes including tumor invasion and metastasis, and angiogenesis [5, 8, 12, 13]. For example, high MMP-2 expression is observed in prostate cancer compared with benign lesions. The MMP-2 expression level was significantly correlated with the tumor grade of prostate cancer [14]. knockdown of MMP9 expression can inhibit breast cancer invasion by increasing cell to cell adhesion and modulating Epithelial-to-Mesenchymal Transition (EMT) genes [15]. MMP-13, is involved in the cleavage of the cell surface receptor TNF-α and the release of ligands. TNF-α can stimulate the metastatic pathway by attracting metastatic factors to the cell surface [16]. Turnover, remodeling, and degradation are the three predominant processes involved in ECM proteolysis [17].

However, the clinical significance of MMPs family in GC have not been completely elucidated. The aim of this study were aimed to clarify the significance of MMPs family expression in GC patients. In order to get convincible results, we first studied MMPs family in The Cancer Genome Atlas (TCGA) database, and then validated it in in-house database. Functional studies also were conducted to known the oncogenesis of related gene.

## RESULTS

### Clinical factors in TCGA and validation cohorts

Table 1 showed the baseline characteristics of the two study cohorts. In TCGA database, there were 360 patients with GC met the selection criteria, including 234 male and 126 female. The median age for all patients was 65 years. 91.11% (328/360) patients had M0 stage. The median length of follow-up was 16 months (range, 1-124 months) and 226 patients had died at the end of follow-up.

**Table 1:** Clinical characteristics of patients with gastric in TCGA and validation cohort

Variable		TCGA		Validated Cohort	
		N	%	N	%
Sex					
	male	234	65.0	91	54.5
	female	126	35.0	76	45.5
Age(Median, Range)		65	30-90	59	51-66
Primary site					
	Antrum/Distal	137	38.1	62	37.1
	Cardia/Proximal	48	13.3	59	35.3
	Fundus/Body	132	36.7	29	17.4
	GEJ	37	10.3	17	10.2
	Unspecific	6	1.7	0	0
Grade					
	G1/G2	133	36.9	71	42.5
	G3	218	60.6	96	57.5
	Gx	9	2.5	/	/
T stage					
	T1	17	4.7	4	2.4
	T2	70	19.4	19	11.4
	T3	167	46.4	85	50.9
	T4	105	29.2	59	35.3
	Tx	1	0.3	0	0
N stage					
	N0	113	31.4	48	28.7
	N1	94	26.1	45	26.9
	N2	72	20.0	37	22.2
	N3	75	20.8	37	22.2
	Nx	6	1.7	0	0
M stage					
	M0	328	91.1	167	100
	M1	18	5.0	0	0
	Mx	14	3.9	0	0

GEJ: Gastroesophageal Junction

There were 167 patients with GC in validation cohort, including 91 male ad 76 female. All patients underwent radical resection, Forty-eight patients with pathological no lymph node metastasis, and the others were lymph nodes positive. After a median follow-up of 32 months (range 1-89 months), 91 out of 167 (54.5%) patients relapsed and 71 out of 167 (42.5%) died from the disease.

### MMP16 were validated as independent predictor for OS in the TCGA cohort

Using univariate Cox regression model, we identified MMP16 was the only predictor for OS of MMPs family in TCGA database (P=0.001, Table 2), Also some other clinicopathological factors, including age at diagnosis (*P*=0.014), primary tumor stage (T stage) (*P* =0.007), node stage(N stage) (*P* =0.001), metastasis stage(M stage) (*P*=0.007) were also found to be high risk factors for OS on univariate Cox proportion hazard ratio analysis (Table 2). Further multivariate Cox regression analysis showed MMP16 expression level was an independent prognostic factor in GC patients. (Hazard ratio (HR): 2.137, 95 % confidence interval (CI): 1.420-3.217, *P* <0.001) (Table 2)

**Table 2:** Univariate and multivariate Cox proportional hazards analysis of Matrix metalloproteinases (MMPs) gene expression and overall survival for patients with gastric cancer in the TCGA cohort

Factor	Univariate analysis		Multivariate analysis	
	HR (95% CI)	P	HR (95% CI)	P
Gender	0.681(0.457-1.016)	0.060		
Age	1.020(0.585-1.792)	**0.014**	1.032(1.012-1.051)	**0.001**
T category	1.375(1.094-1.727)	**0.007**	1.309(1.021-1.679)	**0.034**
N stage	1.300(1.113-1.518)	**0.001**	1.262(1.073-1.485)	**0.005**
M stage	1.590(1.132-2.234)	**0.007**	1.462(1.038-2.059)	**0.030**
Grade	1.313(0.933-1.848)	0.119		
Tumor location	0.971(0.828-1.139)	0.721		
MMP1	1.027(0.948-1.112)	0.516		
MMP2	1.074(0.943-1.223)	0.284		
MMP3	1.006(0.930-1.088)	0.888		
MMP7	1.023(0.946-1.105)	0.569		
MMP8	0.927(0.543-1.582)	0.780		
MMP9	0.969(0.857-1.096)	0.621		
MMP10	1.015(0.875-1.177)	0.847		
MMP11	1.079(0.975-1.194)	0.142		
MMP12	0.952(0.872-1.040)	0.275		
MMP13	1.027(0.855-1.233)	0.775		
MMP14	1.014(0.834-1.231)	0.892		
MMP15	0.931(0.763-1.135)	0.478		
MMP16	1.960(1.311-2.931)	**0.001**	2.137(1.420-3.217)	**<0.001**
MMP17	1.107(0.852-1.437)	0.446		
MMP19	0.972(0.735-1.286)	0.844		
MMP20	1.657(0.722-3.803)	0.233		
MMP21	3.776(0.471-30.257)	0.211		
MMP23A	0.936(0.010-87.788)	0.977		
MMP23B	1.144(0.801-1.633)	0.460		
MMP24	1.097(0.833-1.444)	0.509		
MMP25	0.758(0.566-1.017)	0.064		
MMP26	3.122(0.583-16.707)	0.184		
MMP27	1.995(0.167-23.837)	0.585		
MMP28	0.979(0.846-1.133)	0.781		

Abbreviation: CI, confidence interval; HR, hazard ratio

Bold type indicates statistical significance

### MMP16 expressions level was prognostic factors for both OS and DFS in the validated database

**Figure 1:**
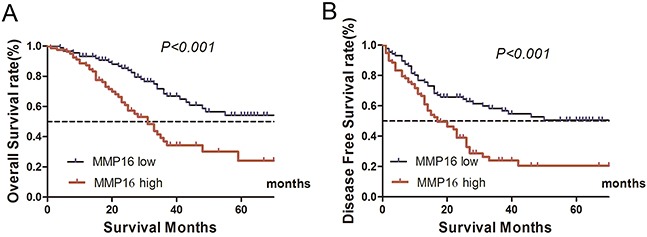
Kaplan–Meier curves depicting OS and DFS in gastric cancer with high and low MMP16 expression High MMP16 expression indicated shorter OS **A.** and DFS **B.** (P<0.05).

For the TCGA database lacks some important clinicopathological factors, such as lymphovascular invasion, perineural invasion and quality of surgery, which may cause confuse in multivariate Cox regression analysis. So, we used an in-house database to validate the results from TCGA database. The expression levels of MMP16 was nearly normal distributed (data not shown), then, we used the median number of MMP16 expression level to divide the patients into low or high risk subgroup. The log-rank test demonstrated that there were significantly higher in the cumulative DFS and OS for patients with low MMP16 expression in tumor tissues than those in high group (both P<0.001; Figure 1).

In a standardized way using a Cox regression model, all factors that were significance in the univariate were tested in multivariate Cox regression analysis for association with OS and DFS. Multivariate analysis demonstrated that MMP16 expression level, T stage, N stage, and tumor grade were independently associated with a decreased OS (*P*<0.05). Similarly, MMP16 expression level, T stage, N stage, and tumor grade were independently associated with a shorter DFS (*P*<0.05) (Table 3 and 4).

**Table 3:** Univariate and multivariate Cox proportional hazards analysis of MMP16 expression and overall survival for patients with gastric cancer in the validation cohort

Factor	Univariate analysis		Multivariate analysis	
	HR (95% CI)	P	HR (95% CI)	P
Gender	1.023(0.641-1.630)	0.925		
Age	1.002(0.983-1.022)	0.828		
T category	2.157(1.475-3.154)	**<0.001**	1.661 (1.078-2.557)	**0.021**
N stage	1.515(1.225-1.874)	**<0.001**	1.211(1.094-1.558)	**0.037**
Grade	1.888(1.147-3.109)	**0.013**	1.668(1.007-2.763)	**0.047**
Lymphovascular invasion	1.545(0.944-2.528)	0.083		
Perineural invasion	1.717(1.031-2.860)	**0.038**	1.626(0.972-2.721)	0.064
Tumor location	1.145(0.930-1.411)	0.202		
MMP16	2.505(1.549-4.050)	**<0.001**	2.069(1.263-3.388)	**0.004**

Abbreviation: CI, confidence interval; HR, hazard ratio

Bold type indicates statistical significance

**Table 4:** Univariate and multivariate cox proportional hazards analysis of MMP16 expression and disease free survival for patients with gastric cancer in the validation cohort

Factor	Univariate analysis		Multivariate analysis	
	HR (95% CI)	P	HR (95% CI)	P
Gender	0.843(0.557-1.275)	0.418		
Age	1.004(0.986-1.022)	0.648		
T category	2.404(1.703-3.394)	**<0.001**	1.954(1.322-2.886)	**0.001**
N stage	1.550(1.287-1.866)	**<0.001**	1.295(1.018-1.648)	**0.004**
Grade	1.721(1.114-2.659)	**0.014**	1.486(0.957-2.308)	**0.078**
Lymphovascular invasion	1.120(0.711-1.766)	0.625		
Perineural invasion	1.487(0.926-2.387)	0.100		
Tumor location	1.006(0.832-1.216)	0.951		
MMP16	2.216(1.450-3.387)	**<0.001**	1.981(1.282-3.058)	**0.002**

Abbreviation: CI, confidence interval; HR, hazard ratio

Bold type indicates statistical significance

### MMP16 exhibited potent oncogenic capacity in GC

**Figure 2:**
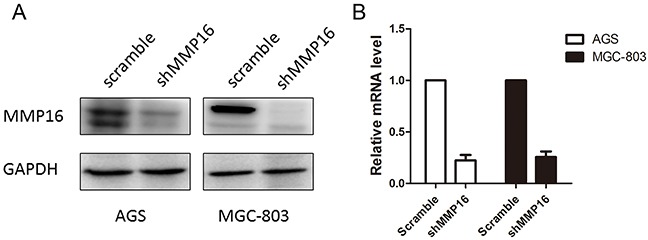
**The knockdown efficiency of shMMP16 was determined by Western blot A.** and RT-PCR **B.**

**Figure 3:**
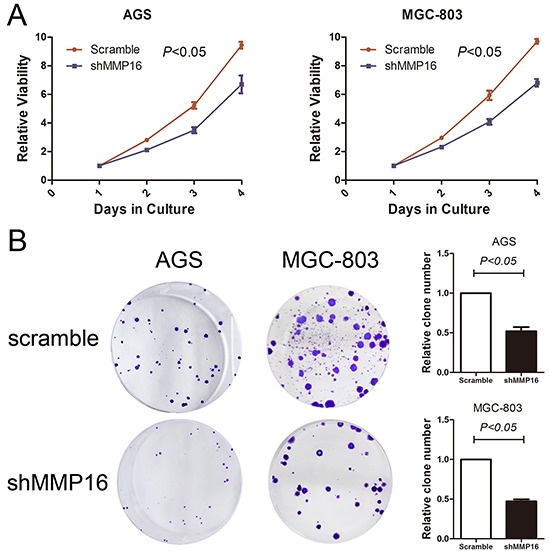
**Silencing of MMP16 expression impaired gastric cancer cell viability determined by CCK8 A.** and clone formation ability **B.** The results are expressed as the mean ± SD of three independent experiments.

To determine the effect of MMP16 on tumorigenesis and progression of colon adenocarcinoma cells, we used lentivirus-mediated silencing GC cell lines, AGS and MGC-803, and the knockdown efficient of the MMP16 was determined by RT-PCR and western blotting (Figure 2). CCK8 analysis demonstrated that cell growth rates in MMP16-shRNA transfected cells were significantly lower than those control cells (P < 0.01, Figure 3). Colony formation assay showed that silencing MMP16 expression lead to a dramatically lower number and smaller colonies (P < 0.01) (Figure 3).

**Figure 4:**
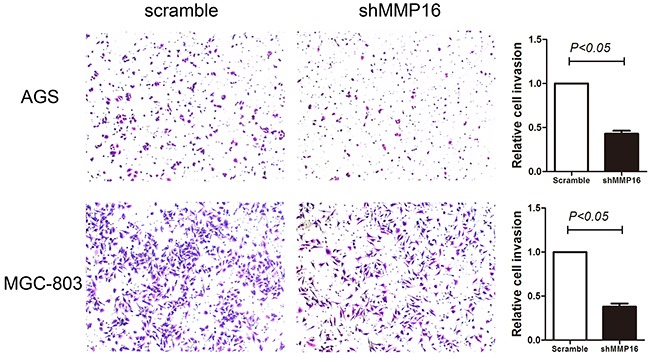
Transwell invasion ability in gastric cancer cells transfected with shMMP16 or scramble Data are presented as the mean ± SD of 3 independent experiments. P <0.05 was considered statistically significant for the controls.

Because high MMP16 expression was statistically correlated with advanced tumor stage in patients’ sample, the impact of MMP16 on GC cells invasion was further investigated. Transwell invasion assay revealed significantly decreased cell invasion with MMP16 silencing (P < 0.05, Figure 4).

## DISCUSSION

Aggressive GC are characterized by early lymph node metastasis or distant metastasis. They always accompanied by a specific gene expression profile, including overexpression of cell-cell adhesion molecules and the membrane-anchored protease. MMPs can degrade extracellular macromolecules at both physiological and pathological conditions. MMPs play critical roles in rheumatoid arthritis, tumor cell invasion, bone resorption, and angiogenesis [18–20]. RNA-sequence have been widely used to screen the candidate genes that may services as new biomarker or target. In the present, we first analyzed the RNA-sequence of GC in TCGA database and found MMP16 (also called MT3-MMP) was an independently prognostic factor for OS in GC. For inherit limitation of TCGA database, we validated such result in in-house database and found high MMP16 expression level was significantly correlated with shorter OS and DFS in GC after radical resection. Functional studies indicated that MMP16 can inhibit GC cell proliferation and invasion.

MMP 16 is a membrane-type metalloprotease located in chromosome 8q21. MMP16 functions in activating pro-MMP2 (gelatinase A) into its active form as the zymogen is excreted out of the cell [21]. Therefore, activating MMP2 would be an indirect mechanism of determining the activity of MMP16 [22]. The activated MMP2 can promote the migration and invasion of tumor cells [20] by denaturing type IV collagen and partially degrading type I collagen and other ECM proteins in basement membrane [23, 24]; Therefore, it is not surprising that high MMP16 expression promoted the invasiveness abilities and lead to poor survival outcomes in GC. Similarly results were reported in some other tumors. For example, increased MMP16 expression mediated a proteolytic switch to facilitate metastasis, and lymphatic invasion and predicted aggressive progression in cutaneous melanoma [25]. Silencing MMP16 expression dramatically inhibit cell migration and invasion of glioma cells [20, 22]. MMP-16 is a downstream of β-catenin target gene in human GC, induction of the MMP16 protein expression is vital to the Wnt-mediated invasive and metastasis in GC cells [25, 26].

Collectively, our data suggest that MMP16 expression is an independent prognostic factor in GC. MMP16 downregulation significantly suppresses cell growth and reduces the invasion abilities in GC cell lines. The frequent upregulation of MMP16 expression in human GC highlights its potential as a novel therapeutic target for this cancer.

## MATERIALS AND METHODS

### Patients in TCGA database

Gene expression data from TCGA stomach adenocarcinoma were downloaded from the website of Cancer Genomics Browser of University of California Santa Cruz (UCSC) (https://genome-cancer.ucsc.edu/). RNA sequencing (RNA-Seq) experiments of TCGA stomach adenocarcinoma had been performed in a combination of two different Illumina platforms (IlluminaGA RNA-Seq and IlluminaHiSeq RNA-Seq datasets) for a total of 300 patients with GC. All patients should have no pretreatment, but with intact overall survival (OS) information. MMP26 and MMP27 were excluded from the study for their mRNA levels were 0 in more than half of the patients (265/360 and 139/360, respectively). Follow-up was completed on Dec 21, 2015.

### Patients in validated database

One hundred and sixty-seven cases of GC diagnosed from January 2005 to December 2008 were obtained to validate the conclusion from TCGA database. All patients were underwent radical surgical resection. None of the patients had received preoperative radiotherapy or chemotherapy. The study was approved by Ethical Committee of Clinical Medical College of Yangzhou University, Subei People's Hospital of Jiangsu Province and performed in accordance with the Declaration of Helsinki (2013) of the World Medical Association. All patients had given written informed consent to the work.

### Real-time PCR

MMP16 mRNA levels were analyzed using a real-time PCR assay. Total RNA was isolated from tissue samples or cultured cells using TRIzol (Invitrogen), and reversely transcripted to cDNA with PrimeScript™ RT Master Mix (Perfect Real Time) kit (RR036A, Takara) based on the manufacturer's instruction. Real-time PCR was performed with the SYBR Green master mix kit using an ABI-7900 thermal cycling instrument (Applied Biosystems). GAPDH gene was amplified as an endogenous control. Primers were as follows: GAPDH-F, 5′- GCA CCGTCAAGGCTGAGAAC-3′, GAPDH-R, 5′-TGGTG AAGACGCCAGTGGA-3′; MMP16-F, 5′- GGACAGAAA TGGCAGCACAAGC -3′, MMP16-R, 5′- CATCAAAGG CACGGCGAATAGC -3′.

### Western blot analysis

Protein levels were evaluated by Western blot. Briefly, cells were lysed in lysis buffer (Pierce, Thermo Scientific, USA) with fresh-added protease inhibitor (Sigma) and the concentration was determined using the BCA protein assay kit (Pierce, Thermo Scientific, USA). Equal amount of protein was separated on sodium dodecyl sulfate polyacrylamide (SDS-PAGE) gel and electro-transferred to a nitrocellulose membrane (Millipore, Billerica, MA, USA). The membrane was blocked with 10% skim milk and the incubated with primary antibodies (MMP6, Abgent, Ap13713b, Wu'Xi, China) overnight at 4°C. The appropriate HRP conjugated secondary antibodies was applied, and membrane was detected with the ECL (Pierce, Thermo Scientific, USA). GAPDH was served as loading control.

### Cell culture

The human gastric cancer cell lines (AGS, MGC-803) were originally purchased from the American Type Culture Collection (Manassas, VA, USA). All cell lines were cultured in medium according to The Defense Technical Information Center recommendation supplemented with 10% FBS (Gibico, Life Technology, Austria).

### Stable transfection of gastric cancer cells

Biologically active short hairpin RNAs (ShRNA) were generated using the lentiviral expression vector pLKO.1-puro. The shRNA target sequence for human MMP16 was 5′- CGTGATGTGGATATAACCATT -3′. PLKO.1-scramble shRNA with limited homology with any known sequences in the human was used as a negative control. AGS and MGC-803 were transfected with the pLKO.1-shMMP16 expression vector or pLKO.1-scramble. The cells stably transfected were isolated using puromycin selection after the cells were transfected with expression vector or control plasmids.

### CCK8

Gastric cancer cells were reseeded into 96-well culture plates at a density of 2 × 10^3^ cells/well and incubated at 37°C. After incubated with 24 h, 10 μl of CCK8 solution was added to each well and incubated for 2 h at 37°C. The plates were detected at 540 nm using a microplate reader (Biotek USA).

### Clone formation assay

6-well plates were seeded with each group of cells at a density of 200 cells per well and cultured for 12 to 14 days. The surviving colonies (>50cells) were counted with crystal violet staining. Colony-forming efficiency (CFE %) was defined as the ratio of the number of colonies formed in culture to the number of cells inoculated.

### Transwell assay

Cell migration assay was performed using Transwell cell culture inserts with 8 μm pores (Corning). The matrigel was added to the inserts for 4 h before cells were plated into inserts. Dissociated cells (5× 10^4^/insert) in serum free medium were seeded on inserts and medium 10 % FBS was added to the lower chambers. After incubation for 36 h, and the non-migrating cells on the upper membrane of insert were erased by cotton swab. The migration cells adhered to the membrane lower surface were fixed with cold 100 % formaldehyde for 20 min, stained with hematoxylin for 25 min and then number of cells was counted under a microscope in five random optical fields.

### Statistical analysis

SPSS (version 19.0; SPSS, Chicago, IL) was used for statistical analysis. All measurement data were presented as mean ± standard deviation (SD) and compared by using the t-test. Enumeration data were presented as percentage or rate, and compared by the chi-square test. All *in vitro* experiments were carried out at least in triplicate. The Kaplan-Meier method was used to analyze gastric cancer patients’ cumulative survival rates. A Cox proportional hazards model was used to calculate univariate and multivariate hazard ratios for the study variables. All P-values were two sided, and < 0.05 was considered statistically significant.
